# Efficacy of Stilbenoid-Enriched Vine Root Extracts in Controlling *Aspergillus carbonarius* Growth and Ochratoxin A Production in Grapes

**DOI:** 10.3390/toxins18090372

**Published:** 2026-08-29

**Authors:** Trang Tran-Minh, Marie-France Corio-Costet, Marie Laurens, Nils Hocquemiller, Jessica Vallance, Marie-Noëlle Bonnin-Verdal, Pierre Waffo-Téguo, Florence Richard-Forget, Vessela Atanasova

**Affiliations:** 1 INRAE, UR 1264 Mycology and Food Safety (MycSA), 33882 Villenave d’Ornon, France; trang.minhtran@ugent.be (T.T.-M.);; 2 Applied Mycology and Phenomics, Department of Plants and Crops, Ghent University, Valentin Vaerwyckweg 1, 9000 Ghent, Belgium; 3 Department of Plant Biotechnology and Bioinformatics, Ghent University, K.L. Ledeganckstraat 35, 9000 Ghent, Belgium; 4 INRAE, Bordeaux Sciences Agro, UMR SAVE 1065, 33882 Villenave d’Ornon, France; 5 Bordeaux INP, Bordeaux Sciences Agro, INRAE, OENO, Univ. Bordeaux, UMR 1366, ISVV, 33140 Villenave d’Ornon, France

**Keywords:** *Aspergillus carbonarius*, ochratoxin A, vine root by-product extracts, stilbenoids, biocontrol

## Abstract

With increased mycotoxin contamination, the search for eco-friendly biocontrol strategies has become increasingly important. This study aimed to evaluate the efficiency of stilbenoid-enriched Merlot (Me) and Tannat (Ta) vine root extracts, and their major active molecule, vitisin B (VIT), against *Aspergillus carbonarius* growth and its ochratoxin A (OTA) production using in vitro liquid and solid, and ex vivo grape-berry assays. In liquid assay, Me and Ta (125 mg/L) and VIT (36 mg/L) significantly inhibited extracellular OTA. Additionally, VIT temporarily reduced both fungal growth and total OTA. Transcriptomics showed that VIT and Me slightly induced OTA biosynthesis genes. In solid and ex vivo assays, doses above 1 g/L decreased OTA production by up to 48% without impacting fungal growth. This lack of correlation suggested a distinct regulatory response to extract-induced stress. Importantly, the fungicide tebuconazole showed method-dependent effects. On in vitro solid medium, it significantly inhibited the fungal growth without affecting OTA yield, whereas in ex vivo berry assays, it significantly inhibited both. Our findings emphasize the potential of stilbenoid-enriched extracts as biocontrol agents targeting OTA production, highlighting the value of multi-assay evaluations. Future studies should elucidate their modes of action and optimize their efficacy.

## 1. Introduction

Fungal pathogens are major causal agents of plant diseases and represent one of the most diverse groups of significant ecological and economic threats. Among the fungal genera most frequently associated with crop damage and mycotoxin contamination are *Aspergillus*, *Fusarium*, and *Penicillium*. The genus *Aspergillus* can affect a large range of agricultural crops during field cultivation or storage, including maize, peanut, onion, garlic, coffee, spices, cocoa, and dried fruits. It is commonly considered as the most worrying genus of toxigenic fungi [1]. Grapes, one of the most important fruit crops worldwide, cultivated mainly for wine production, are frequently contaminated by fungi belonging to the genus *Aspergillus* section *Nigri* (commonly called black *aspergilli*). The predominant black *Aspergillus* species include *Aspergillus niger*, *Aspergillus carbonarius*, *Aspergillus aculateus*, *Aspergillus japonicus*, *Aspergillus uvarum*, *Aspergillus tubingensis, Aspergillus welwitschiae,* and *Aspergillus awamori* [2]. Some of these fungi are capable of producing the ochratoxin A (OTA) mycotoxin. OTA is a polyketide-derived metabolite, composed of a chlorinated type I polyketide dihydroisocoumarin moiety linked to L-phenylalanine. Its biosynthesis is associated with a gene cluster that contains *otaR1* (encoding a basic leucine zipper (bZIP) transcription factor), *otaA* (polyketide synthase), *otaB* (nonribosomal peptide synthetase), *otaC* (cytochrome P450 monooxygenase), *otaD* (halogenase), *otaY* (cyclase), and *otaR2* (encoding a zinc finger DNA binding protein) [3,4,5].

Among fungal species isolated from grapes, *A. carbonarius* is the primary one responsible for OTA contamination. As indicated by Battilani and Camardo-Leggieri [6], almost 100% of *A. carbonarius* isolates are strong OTA producers, in contrast with 5–10% for the *Aspergillus niger* species. OTA is classified by the International Agency for Research on Cancer (IARC) in group 2B, i.e., as a possible human carcinogen. In addition, this toxin exhibits nephrotoxic, hepatotoxic, teratogenic, neurotoxic, genotoxic, and immunotoxic properties [7,8,9]. The awareness that OTA can have serious deleterious effects on both humans and animals has led many countries to establish regulations limiting its presence in food and feed. In Europe, Regulation (EC) No. 1881/2006 sets maximum permissible levels of OTA in various foodstuffs.

OTA can be found in table grapes, as well as in grape products such as wine, grape juice, and dried vine fruit. It is established that wine consumption is the second most frequent source of OTA in European diet [10]. In the survey published by Valero et al. [11], analysis of European wines revealed that approximatively 20% exceeded the EU’s maximum OTA limit of 2 µg/kg. More recently, Ortiz-Villeda et al. [12] analyzed wine reports from 2012 to 2020 and highlighted that the occurrence of OTA varied markedly depending of the harvest year. The previous authors also warned of an increase in OTA levels in wines since 2014. Environmental conditions strongly influence mycotoxin production. OTA contamination in grapes varies significantly across season to season and is strongly related to geographical area, microclimate, weather conditions, and plant phenology [13,14].

In this context, developing sustainable and eco-friendly strategies for controlling *A. carbonarius* and associated OTA contamination is of major importance, not only to ensure the safety and quality of agricultural commodities but also to limit the excessive use of synthetic fungicides in accordance with the ambition of the European Green Deal. Several investigations revealed natural products and microorganisms possessing inhibiting properties against OTA production and/or *A. carbonarius* growth. These include various microorganisms such as rhizobacteria, *Bacillus subtilis*, *Paenibacillus alvei*, and several yeast species [15,16,17,18]. For example, iturin A was identified as the metabolite responsible for antifungal activity of *B. subtilis*. This compound inhibits *A. carbonarius* by downregulating genes associated with cell membrane integrity, transport functions, energy metabolism, oxidation–reduction processes, and osmotic regulation [16]. As documented in various reports [8,19,20], natural compound-based biofungicide formulations, particularly those rich in essential oils and phenolic compounds, could be part of such eco-friendly strategies. Notably, stilbenoids that are a class of naturally occurring phenolic compounds present in a variety of plant species, including grapevine (*Vitis vinifera*), could be seen as a promising option to reduce synthetic fungicide [21,22]. In grapevine, these compounds act as phytoalexins elicited by various biotic and abiotic agents, including *A. carbonarius* infection [23]. Indeed, several studies have reported the ability of resveratrol and its monomeric, dimeric, and prenylated derivatives to inhibited the growth and/or OTA production by various *Penicillium* and *Aspergillus* species [24,25,26]. Cai et al. [22] suggested that the stilbene content of berries may regulate OTA production in grapes infected with *A. carbonarius*. Docking analyses further indicated that some stilbenes, such as pterostilbene, bind more tightly to specific *A. carbonarius* enzymes, which could explain their antifungal and OTA-inhibiting properties.

A few studies, summarized in a review by Tran et al. [21], have also addressed the antifungal and antimycotoxin capacities of stilbenoids derived from agro-wastes against mycotoxigenic fungi. In a recent work, extracts obtained from the canes, wood, and root of five grapevine varieties, were described as efficient to inhibit fungal growth and reduce mycotoxin production by *Fusarium graminearum*, the most active extracts being Merlot (Me) and Tannat (Ta) root extracts [27,28]. Use of high-resolution mass spectrometry data has allowed the previous authors to determine that stilbenoid oligomers, especially vitisin B (VIT), were the main contributors of the antifungal and antimycotoxin effects of the tested stilbenoid extracts from grapevine by-products [27,28]. The inhibitory effects of stilbenoid-rich cane extracts against various toxigenic species and their mycotoxins have also been recently corroborated by Aznar et al. [29].

Using a multi-omics strategy combining transcriptomics and metabolomics, Tran-Minh et al. [30] have partially deciphered the mode of action of the stilbenoid tetramer VIT underlying its antifungal and antimycotoxin activity against *F. graminearum*. Three pivotal effects were demonstrated: (1) damage in the fungal cell wall and plasma membrane by interfering with sphingolipid biosynthetic pathway, (2) inhibition of the sporulation and hyphal growth, and (3) direct and indirect reduction in the biosynthesis of type B trichothecene mycotoxins.

Given the strong biological activities of the stilbenoid-enriched root extracts from Merlot and Tannat, as well as their major stilbenoid, VIT, against *F. graminearum*, the present study aims to evaluate their antifungal and antimycotoxin effects on the growth and OTA production of *A. carbonarius* using a sterol biosynthesis inhibitor fungicide as positive control on solid media. Various approaches, ranging from in vitro assays to ex vivo assays on grape-berries, were used; results, advantages, and disadvantages associated with this set of methodologies were discussed with the aim to provide some suggestions to guide the screening of natural molecules for biocontrol applications. Particular attention was also given to the stilbenoid composition of the extracts and its relationship with their bioactivities in order to better explain the observed effects.

## 2. Results

### 2.1. Effect of Pure Stilbenoid VIT and Stilbenoid-Enriched Extracts on the Growth and OTA Production of Aspergillus carbonarius in Liquid Medium

**Figure 1:**
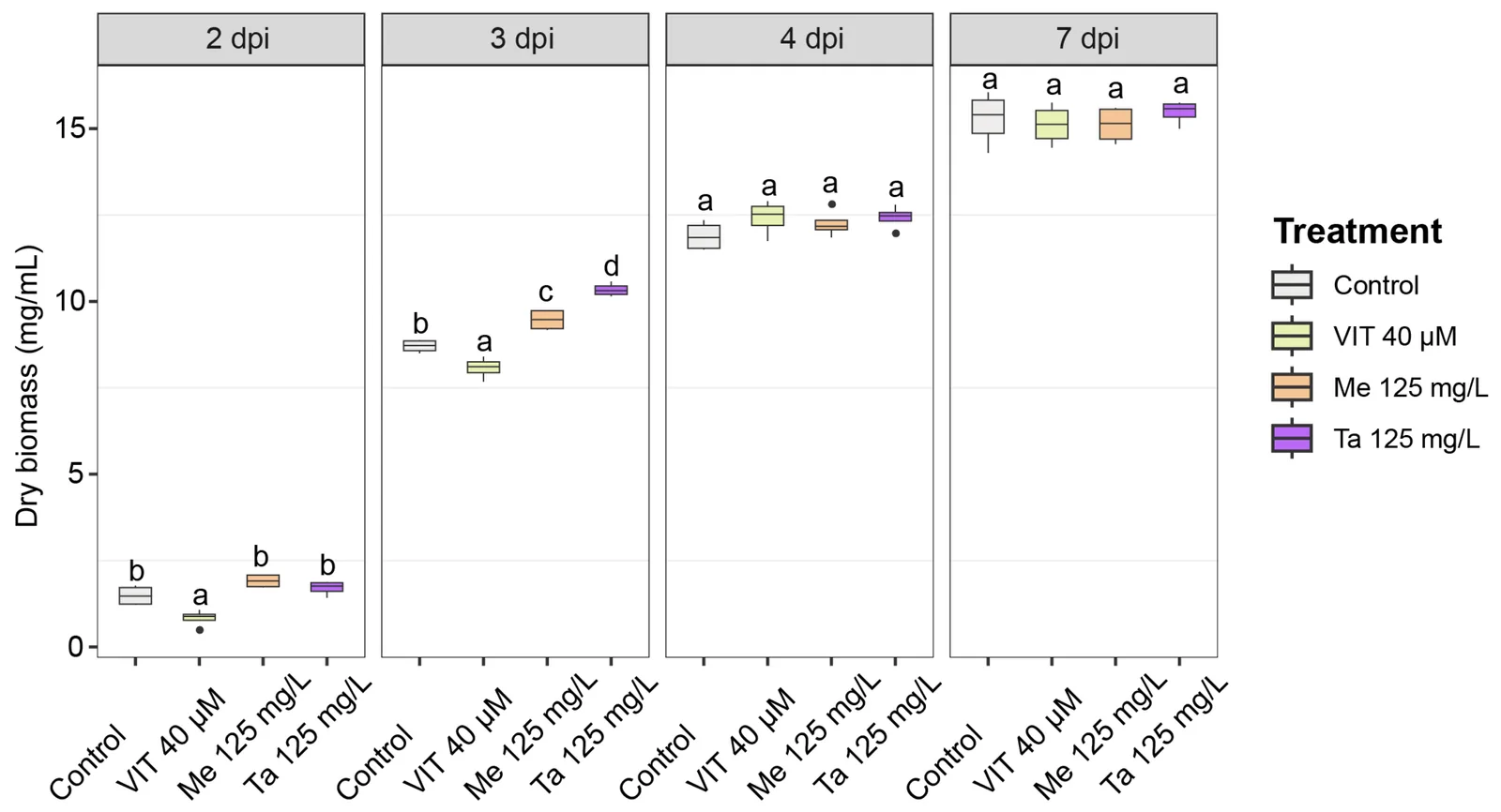
Kinetics of *Aspergillus carbonarius* ATCC-4641 growth in CYA liquid media supplemented with 40 µM (36.28 mg/L) vitisin B (VIT), 125 mg/L stilbenoid extract from Merlot roots (Me), or 125 mg/L stilbenoid extract from Tannat roots (Ta). Ethanol 0.5% was used as the control. Different letters indicate a statistically significant difference between treatments at each time point (*p* < 0.05). Four biological replicates were performed. dpi: days post inoculation.

**Figure 2:**
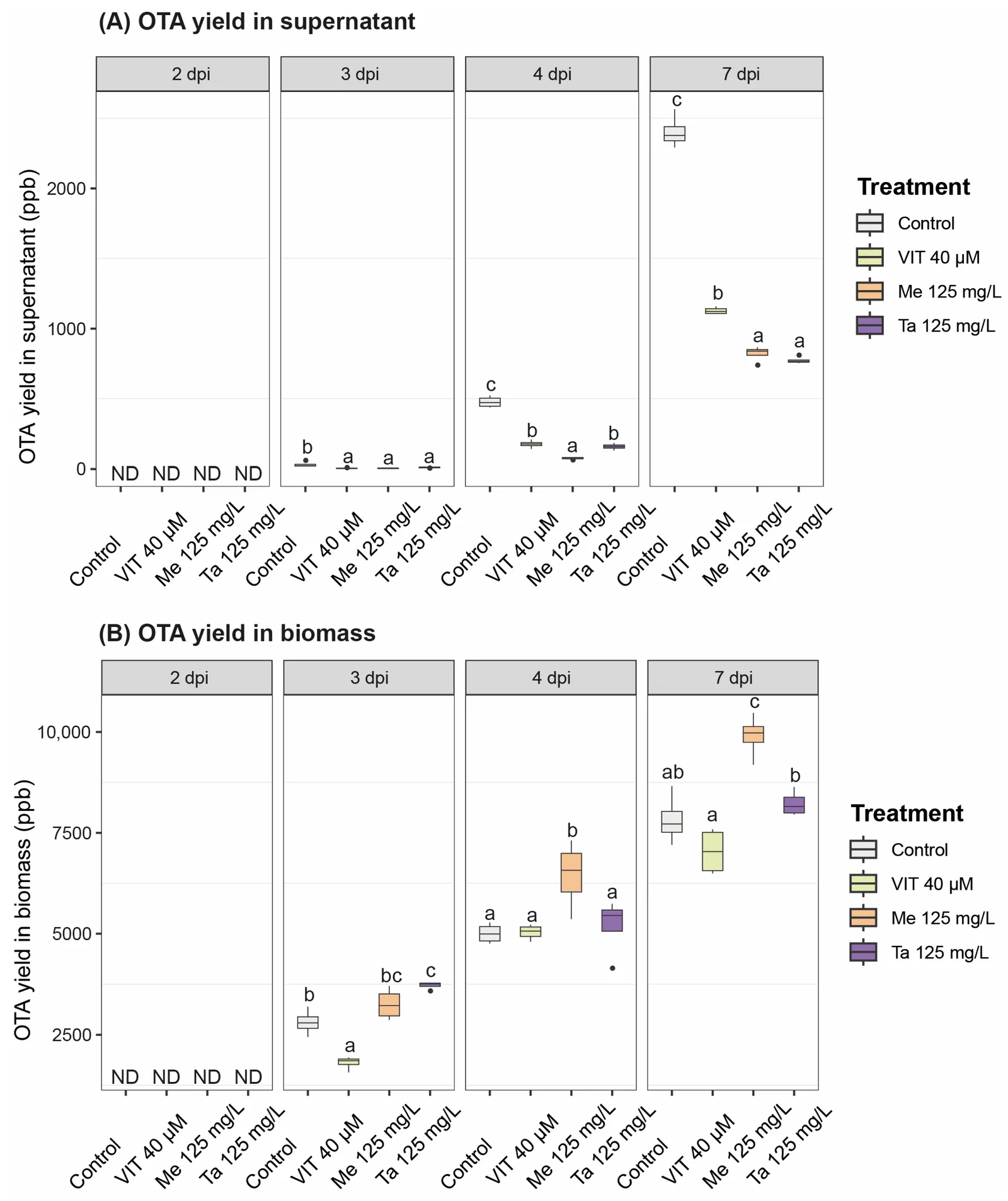
Kinetics of ochratoxin A (OTA) accumulation in (**A**) supernatants and in (**B**) mycelia of *Aspergillus carbonarius* ATCC-4641 supplemented or not with 40 µM (36.28 mg/L) vitisin B (VIT), 125 mg/L stilbenoid extract from Merlot roots (Me) or 125 mg/L stilbenoid extract from Tannat roots (Ta). Ethanol 0.5% was used as the control. Different letters indicate a statistically significant difference between treatments at each time point (*p* < 0.05). Four biological replicates were performed. dpi: days post inoculation. ND: not detected.

VIT (40 µM or 36.28 mg/L) and Me and Ta extracts (both at 125 mg/L) were supplemented in CYA (Czapek Yeast Autolysate) liquid cultures in the presence of *A. carbonarius* ATCC-4641. These concentrations were selected according to preliminary tests and their solubility in the studied medium. Fungal growth and OTA accumulation were monitored for up to 7 days post inoculation (dpi) (Figure 1 and Figure 2). As shown in Figure 1, VIT significantly inhibited fungal growth at 2 and 3 dpi. By contrast, at 2 dpi, Me and Ta had no effect; however, at 3 dpi, both stilbenoid extracts significantly stimulated fungal growth compared to the untreated control, resulting in higher dry biomass. These effects diminished over time, and at 4 dpi, no statistically significant differences were detected in any condition, a trend that persisted up to 7 dpi. Concerning VIT, these results suggested the modifications induced during the early stages of fungal development corresponded to a delayed effect of inhibition that faded over time.

Regarding OTA accumulation in supernatants (Figure 2A), the toxin was first detected 3 days post inoculation. OTA levels increased between 3 and 7 dpi, reaching approximately 30, 477, and 2402 ppb in control cultures at 3, 4, and 7 dpi, respectively. VIT, Me, and Ta significantly inhibited OTA production at all stages of fungal development. In VIT-treated cultures, the relative reduction in OTA decreased over time, reaching 85%, 63%, and 53% at 3, 4, and 7 dpi, respectively, compared to the corresponding controls. By contrast, the inhibitory effect of Me and Ta remained stable over time, with reduction of 84% for Me and 67% for Ta (Figure 2A). Stilbenoid extracts reduced OTA concentrations in the supernatants more strongly than VIT.

When measured in mycelia, OTA was first detected in 3-day-old mycelia (Figure 2B) and increased almost linearly between 3 and 7 dpi, reaching approximately 2809, 5007, and 7824 ppb in control cultures at 3, 4, and 7 dpi, respectively. It is important to emphasize that OTA concentrations were markedly higher in the mycelia than in the corresponding supernatants, with levels approximatively 94-, 10-, and 3-times greater at 3, 4, and 7 dpi, respectively.

As shown in Figure 2B, VIT, Me, and Ta exhibited different effects on OTA production by *A. carbonarius* ATCC-4641 over time. At 3 dpi, VIT significantly inhibited OTA production (by 36%, *p*-value < 0.05), but had no inhibitory effect at 4 and 7 dpi. By contrast, at 3 dpi, Ta significantly stimulated OTA biosynthesis by 32%, with no effect thereafter. The Me extract was the only extract that progressively enhanced the toxin production by 16%, 29%, and 27% at 3, 4, and 7 dpi, respectively.

### 2.2. Effect of VIT and Stilbenoid-Enriched Extracts on the Expression of Genes Involved in OTA Biosynthetic Pathway in Aspergillus carbonarius ATCC-4641 in Liquid Medium

To understand why VIT and the stilbenoid extracts reduced extracellular OTA levels in the supernatant but did not significantly affect intracellular OTA in the biomass, we further examined their effects on the expression of key genes involved in the *A. carbonarius* OTA biosynthetic pathway. These genes include *otaA* (polyketide synthase), *otaB* (nonribosomal peptide synthetase), *otaC* (cytochrome P450 monooxygenase), *otaD* (halogenase), and *otaY* (cyclase) [3,4,5].

**Figure 3:**
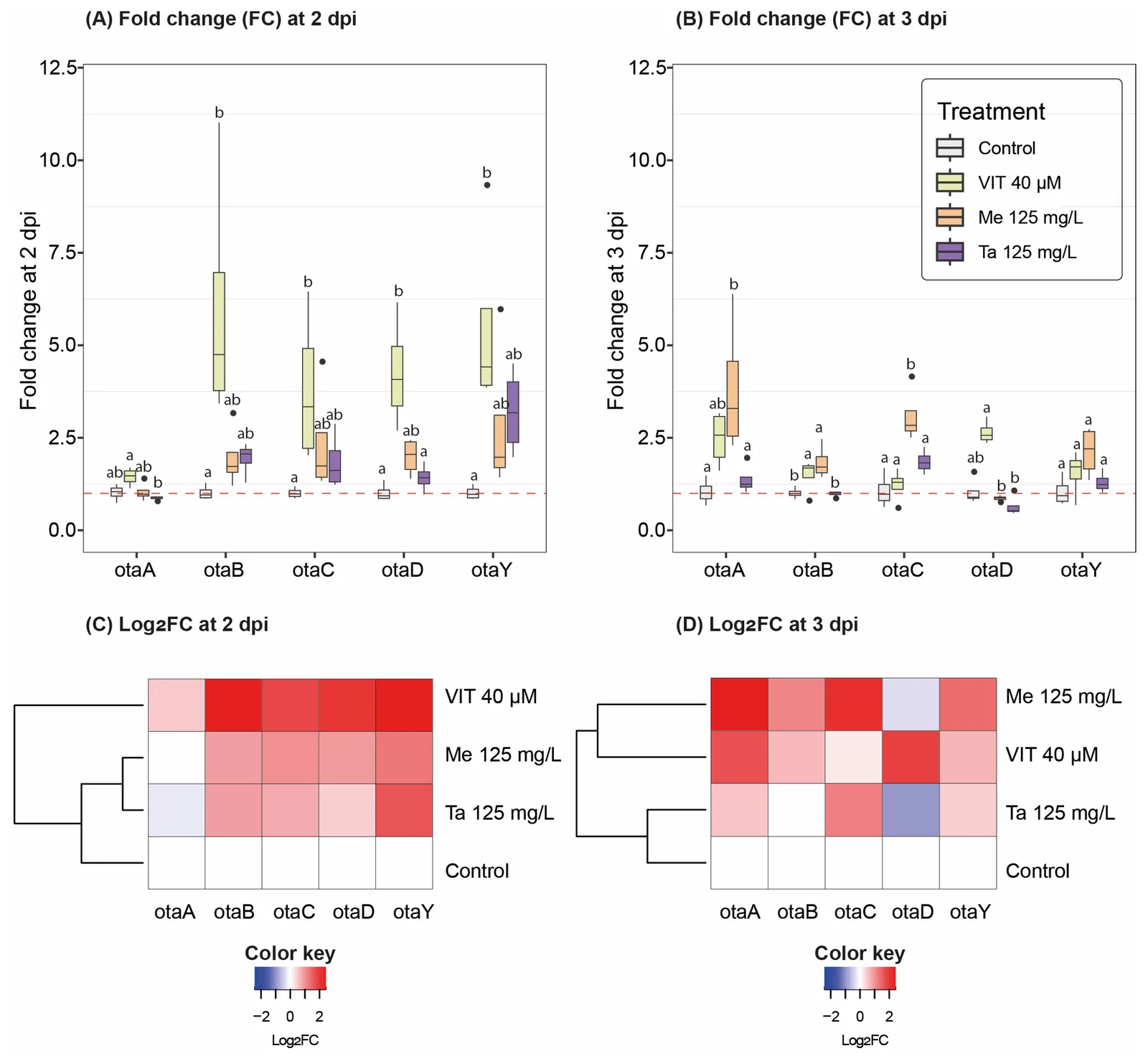
Effects of vitisin B (VIT), stilbenoid extract from Merlot roots (Me) or stilbenoid extract from Tannat roots (Ta) on expression levels of genes involved in ochratoxin A (OTA) biosynthetic pathways in *Aspergillus carbonarius* ATCC-4641. The gene expression was observed at 2 timepoints, 2- and 3- days post inoculation (dpi), and expressed as fold change (**A**,**B**) and log_2_FC (**C**,**D**). Ethanol 0.5% was used as control. Different letters indicate a statistically significant difference between treatments at each time point (*p* < 0.05). Four biological replicates were performed. The lines on the left of the heat map represent the hierarchical clustering between treatments. The dash red lines are the threshold of fold change of 1.

To assess gene expression, mycelia were collected at the early time points of 2 and 3 dpi following exposure to VIT and the two extracts Me and Ta. Targeted transcriptomics was performed using the primers listed in Table S1. The gene expression results are presented in Figure 3.

At 2 dpi, VIT significantly induced the upregulation of OTA-related genes, except for *otaA*, compared to the untreated control (Figure 3A,C). This is evidenced by the fold-change (FC) values for *otaB* (FC = 6.0, *p* < 0.05), *otaC* (FC = 3.8, *p* < 0.05), *otaD* (FC = 4.3, *p* < 0.05), and *otaY* (FC = 5.5, *p* < 0.05). A similar, although less pronounced, trend was observed for the Me and Ta extracts; however, these changes were not statistically significant compared to the control (Figure 3A).

At 3 dpi, the VIT-induced upregulation of *otaB* persisted, while the expression of *otaC*, *otaD*, and *otaY* was no longer significantly different from the control. The Me extract showed significant induction of *otaA* (FC = 3.8, *p* < 0.05), *otaB* (FC = 1.8, *p* < 0.05), and *otaC* (FC = 3.1, *p* < 0.05), but had no significant effect on *otaD* or *otaY*. By contrast, the Ta extract did not significantly affect the expression of any of the OTA-related genes. Although *otaD* was slightly downregulated by Ta (log_2_FC = –0.7), this change was not statistically significant (Figure 3B,D).

In summary, the data demonstrate that *A. carbonarius* tends to upregulate OTA biosynthesis genes when exposed to VIT and the stilbenoid extracts, possibly as part of a stress-response mechanism.

### 2.3. Effect of Pure Stilbenoid VIT and Stilbenoid-Enriched Extracts on the Growth and OTA Production by Aspergillus carbonarius in Solid Medium

**Figure 4:**
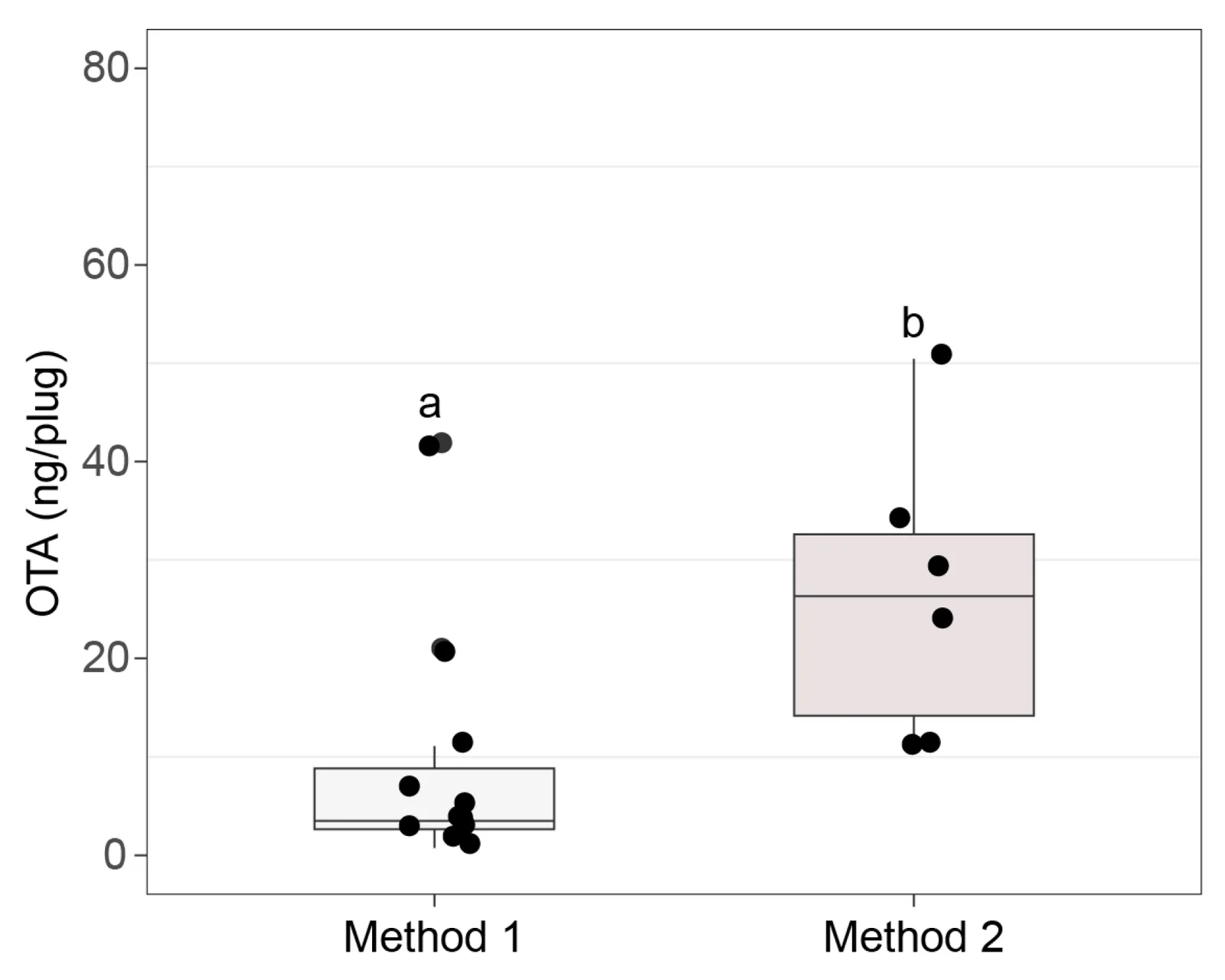
Amounts of ochratoxin A (OTA) expressed in ng/plug, produced by *A. carbonarius* strains ATCC-4641 depending on the method used. Method 1: spores were spread on solid medium and then sprayed with the product or ethanol 0.7% as control. Method 2: spores were mixed with the product or ethanol 0.7% and 3–5 drops of the mixture were deposited on the medium. Different letters indicate a statistically significant difference (*p* < 0.05).

In this section, the effects of VIT, Me, and Ta extracts, as well as the fungicide tebuconazole on fungal development and OTA production were evaluated on a solid medium, using ATCC-4641 *A. carbonarius* strain. Fungal development was assessed by counting the germinated spores on Petri dishes when Method 1 was used, and by measuring colony diameter (mm) when Method 2 was applied as described in Section 5 (Figure S1). The two methods were compared in terms of OTA production by ATCC-4641 strain. A statistically significant difference in OTA production was observed (Figure 4). Method 1 exhibited greater variability, as reflected by a larger standard deviation. OTA levels reached 10.8 ± 15.5 ng/plug using Method 1 and 26.1 ± 11.5 ng/plug using Method 2.

#### 2.3.1. Effect of the Fungicide Tebuconazole on the Fungal Growth and OTA Production by *Aspergillus carbonarius* ATCC-4641 Strain

**Figure 5:**
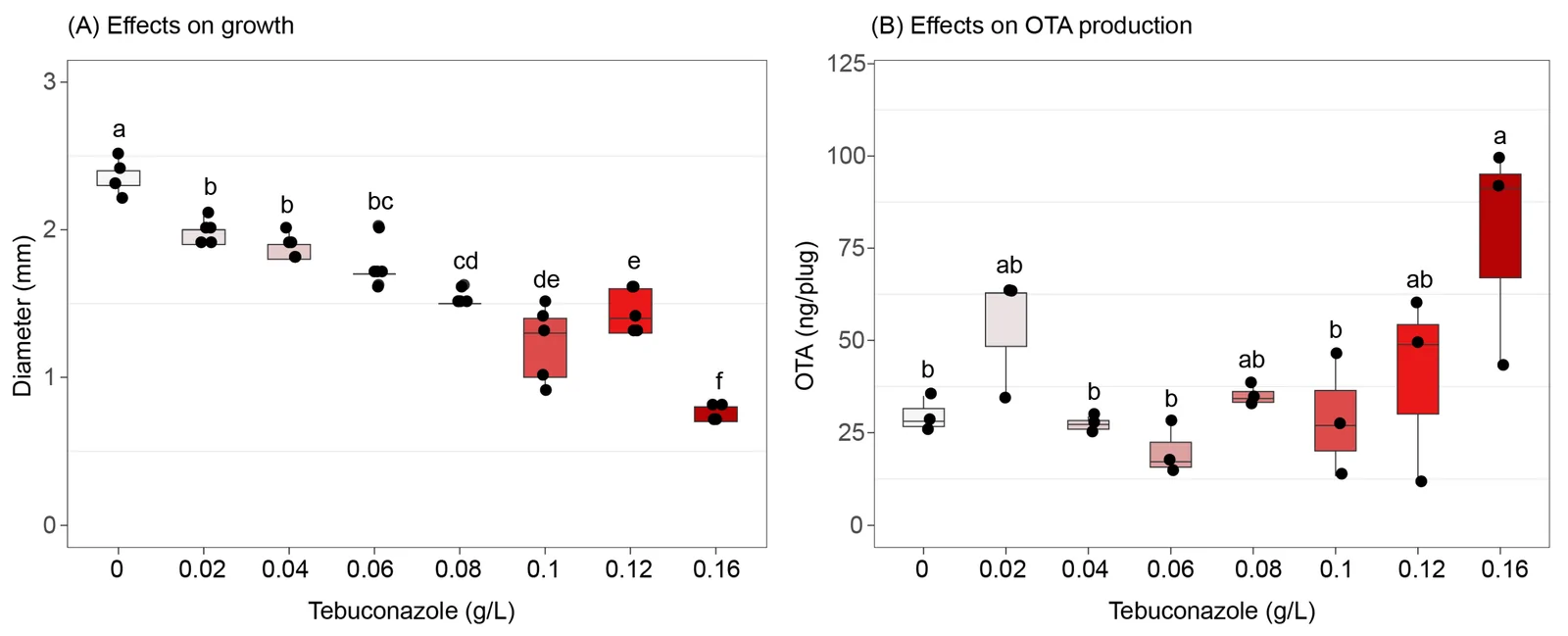
Effect of tebuconazole at concentrations ranging from 0.02 to 0.16 g/L on *A. carbonarius* strain ATCC-4641, (**A**) fungal growth and (**B**) ochratoxin A (OTA) production. Method 2 was used involving the mixing of spores with tebuconazole or ethanol 0.7% prior to the deposition of 3–5 drops of the mixture onto the medium. Different letters indicate a statistically significant difference (*p* < 0.05).

To determine whether fungal growth and OTA production are positively correlated in response to fungicide treatment, we evaluated the effect of tebuconazole at different concentrations on the fungal growth and OTA production by *A. carbonarius* ATCC-4641 strain using in vitro solid medium assays. As illustrated in Figure 5A, this fungicide displayed a dose-dependent effect with increasing growth inhibition at higher concentrations. At the highest concentration tested (0.16 g/L), fungal growth inhibition reached 68.4% in comparison with control condition. However, tebuconazole did not reduce OTA production. Instead, at 0.16 g/L, it induced a significant 2.7-fold increase in OTA production compared to the control (Figure 5B). These findings suggest that there was no positive correlation between fungal inhibition and OTA production by *A. carbonarius* in response to fungicide treatment on solid medium.

#### 2.3.2. Effect of Pure Stilbenoid VIT and Stilbenoid-Enriched Extracts from Merlot and Tannat Roots on Fungal Development and OTA Production by *Aspergillus carbonarius*

To investigate the effect of VIT, Me, and Ta on *A. carbonarius* and its OTA production by the ATCC-4641 strain, each treatment was tested at a concentration of 1 g/L. This concentration was higher than that used in the liquid assays because these extracts showed only limited inhibitory effects at 125 mg/L in the liquid assays, necessitating a higher concentration to evaluate their activity under solid-state conditions.

The results revealed that fungal development of the *A. carbonarius* strain was not significantly affected by Me, Ta, and VIT compared with the corresponding controls (Table S2).

**Figure 6:**
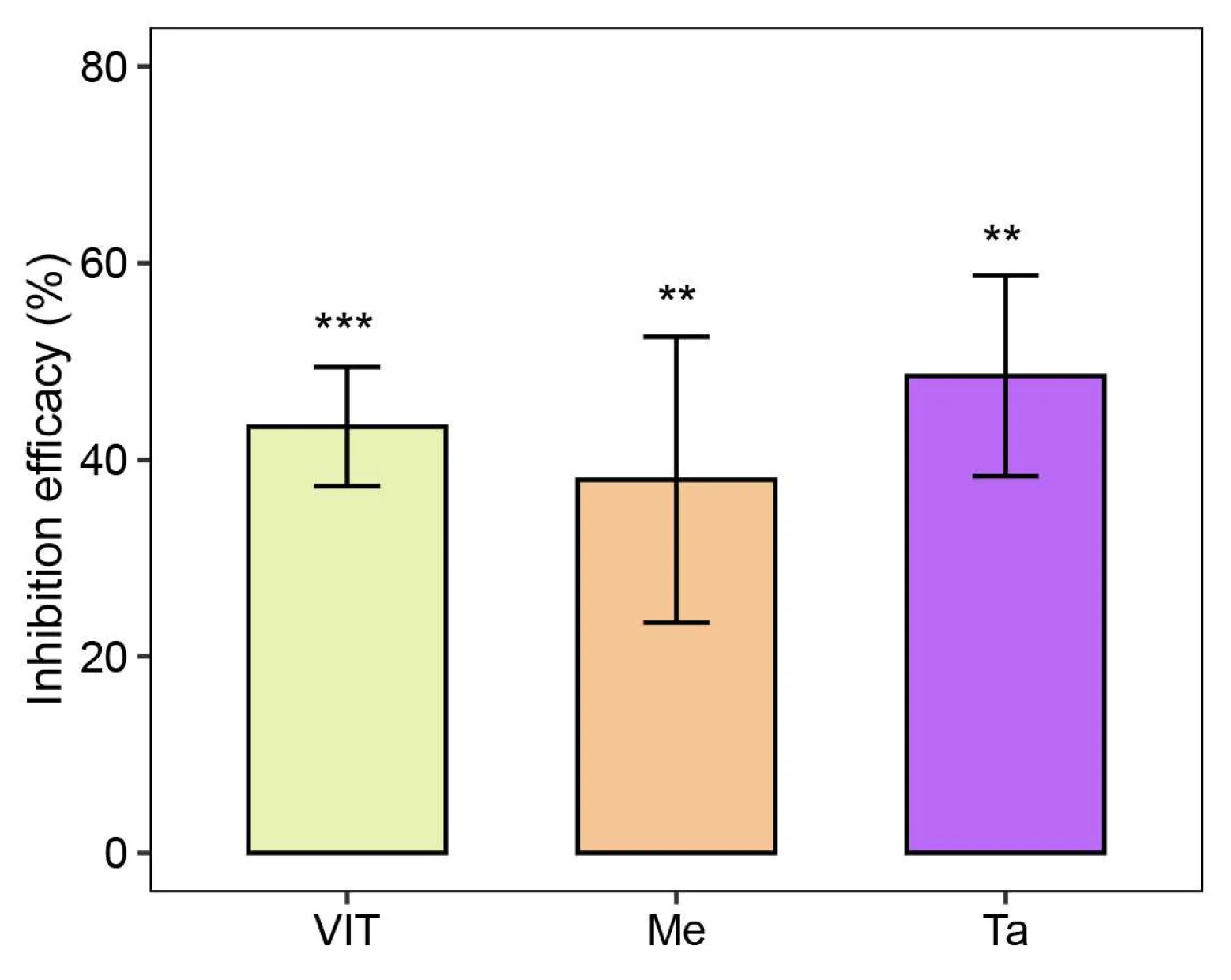
Inhibition efficacy of Merlot (Me) and Tannat (Ta) stilbenoid-enriched root extracts and of vitisin B (VIT) at 1 g/L on ochratoxin A (OTA) production by ATCC-4641 strain compared to the corresponding control on the solid medium. Method 1 was used for the application of the spores and the treatments: the spores were spread on the solid medium and then sprayed with products or ethanol 0.7% for the control condition Asterix indicate a statistically significant difference when compared with corresponding control (** *p* < 0.01, and *** *p* < 0.001). For VIT, the data were based on three biological replicates, and for extracts, the data were based on four independent experiments, each performed in triplicate. The inhibition efficacy was calculated according to the following formula: inhibition efficacy, % = [(OTA concentration in control culture − OTA concentration in treatment-amended culture)/OTA concentration in control culture] × 100.

The inhibition efficacy of the two stilbenoid-enriched extracts and VIT on OTA production is reported in Figure 6. All treatments significantly reduced OTA accumulation compared with the corresponding control. The highest inhibition rate was observed for Ta (48%), followed by VIT (43%) and Me (38%).

In summary, using in vitro solid medium assays, we found that VIT and the extracts at higher doses of 1 g/L significantly suppressed the OTA production by the *A. carbonarius* ATCC-4641 strain by up to 48% without affecting fungal development. These findings contrast with those obtained for tebuconazole, where increasing concentrations of the fungicide resulted in a gradual, dose-dependent growth inhibition alongside a significant spike in OTA levels at the highest concentration (0.16 g/L).

### 2.4. Ex Vivo Assessment of the Impact of Stilbenoid-Enriched Extract from Merlot Roots on Aspergillus carbonarius Fungal Development and OTA Production in Grape-Berries

Data on the levels of OTA detected in untreated grape-berries varied between 55.8 and 167.6 µg/berry, values that were slightly higher than those obtained in the solid medium.

**Figure 7:**
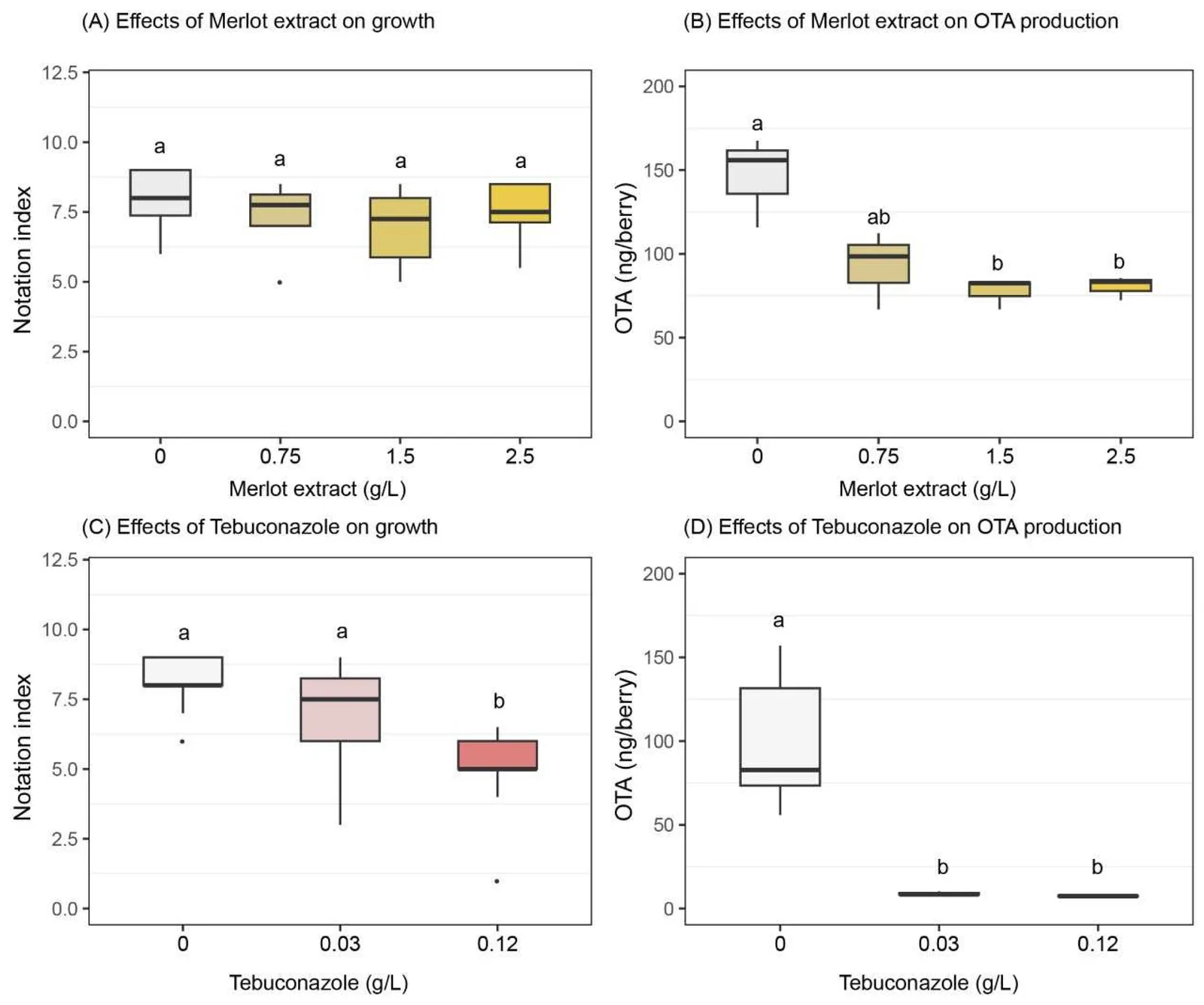
Effect of (**A**,**B**) stilbenoid-enriched extract from Merlot roots (Me) and (**C**,**D**) tebuconazole at different concentrations on fungal development and OTA production on grapes berries inoculated with *A. carbonarius* ATCC-4641 strain. Different letters indicate a statistically significant difference (*p* < 0.05). For fungal development, the data were based on nine biological replicates, and for OTA quantification, the data were based on three biological replicates.

Ex vivo effects of the Me extract on fungal development and OTA production were investigated for ATCC-4641 strain and compared with the effects of the fungicide (Figure 7 and Figure S2). At the tested concentrations of 0.75, 1.5, and 2.5 g/L, Me extract had no effect on the fungal growth (Figure 7A). Tebuconazole, on the other hand, significantly inhibited the development of the ATCC-4641 strain at a concentration of 0.12 g/L (Figure 7C).

Regarding OTA production, the Me extract significantly inhibited toxin production by 47% and 45% at concentrations of 1.5 and 2.5 g/L, respectively. No differences were observed with 0.75 g/L. (Figure 7B). As shown in Figure 7D, at the tested concentrations of 0.03 and 0.12 g/L, tebuconazole significantly inhibited OTA production by more than 90%.

## 3. Discussion

In response to the growing societal demand for alternatives to chemical fungicides, biocontrol strategies based on the valorization of agricultural by-products is a promising option to develop environmentally friendly solutions for protecting plants against fungal pathogens. In France, it is estimated that around 8 million tons of viticultural wood waste (incl. 1.8 million tons of canes and 6 million tons of wood and roots) are generated annually [28]. Multiple studies indicate that these by-products are rich natural sources of stilbenoids [31,32], a class of phenolic compounds with biological activity against toxigenic pathogens, including those affecting grapevines [21,33,34,35]. However, their antimycotoxin activity remains poorly investigated. Based on the notable biological activities of the stilbenoid tetramer-enriched root extracts from Merlot and Tannat, as well as the pure stilbenoid tetramer VIT against *F. graminearum* [27,30], the aims of the present study were: (i) to investigate their antifungal and antimycotoxin effects on *A. carbonarius* and its OTA production, and (ii) to assess the effectiveness and relevance of different methods used in the key stage of selecting natural extracts as biocontrol agents. The stilbenoid composition of the studied extracts is presented in Table 1. Nine compounds were quantified, including two monomers (resveratrol and piceatannol), four dimers (ε-viniferin, ω-viniferin, pallidol and ampelopsin A), two trimers (α-viniferin and miyabenol C), and one tetramer (R-viniferine named also vitisin B) [27]. The two extracts were selected because of their high concentration of the VIT tetramer, reaching 113 and 125 mg/g in Me and Ta, respectively. Compared with Me, the Ta extract exhibited both greater qualitative diversity and higher overall stilbenoid content. It contained approximately three-fold higher levels of monomers, two-fold higher levels of dimers, and additionally contained the trimer miyabenol C, which was not detected in Me.

In this work, three biological methods, commonly used by the scientific community—liquid medium, solid medium, and grape-berry assays [27,29,36,37,38], were applied to evaluate the efficacy of VIT, Me and Ta extracts, as well as tebuconazole, in preventing the fungal growth of *A. carbonarius* and its OTA production. Given that the product concentrations in the solid and grape-berry assays are 6 to 20 times higher than in the liquid medium, these methodologies are not directly comparable; therefore, a comparative summary of their advantages and limitations was provided (Table 2). Overall, liquid and solid medium assays are simple, inexpensive, and easy to implement. Liquid medium assays allow straightforward biomass quantification and require only small amounts of the tested products, since the fungal cells are fully immersed in the products, often resulting in higher apparent efficacy. In addition, tests in liquid medium yielded the most reproducible results and have therefore been recommended as a standard method for evaluating the bioactivity of natural products [39,40]. Experiments conducted on solid media and grape-berries require substantially larger amounts of the tested product, 10–20 times higher than those used in liquid medium. This increase is primarily due to the mode of administration: spores are either mixed directly with the product during deposition or are exposed to it through spraying onto pre-adsorbed spores. Additionally, adsorption phenomena on solid surfaces and restricted diffusion lead to higher localized concentrations of the product, further enhancing uptake and resulting in greater overall consumption compared with liquid conditions. While fungal biomass can be quantified on a solid medium using a cellophane film, the berry method does not allow reliable quantification unless qPCR is employed.

On a solid medium, the effective contact time between the extract solution and the fungus appears to be reduced, which may limit the product’s efficacy. Furthermore, as the extracts were administered in an aqueous hydroalcoholic solution, their bioavailability is likely to be low. Appropriate formulation strategies could improve penetration and enhance the overall efficacy of the extracts at the tested concentrations. On grape-berries, the spores are initially exposed to the extracts; however, as the fungus grows and utilizes the berry’s nutrients, the effective dose becomes progressively diluted over time. The grape-berry assay represents the system that most closely reflects real-world conditions, but it presents several constraints. To ensure the reproducibility of product testing, it is necessary to obtain decontaminated, untreated berries with similar physiological characteristics. In addition, the method is limited by the seasonal availability of grapes. Nevertheless, OTA production appears to be higher on berries, possibly due to greater environmental water availability [41,42]. The higher nutrient richness of berries compared with MEA medium, may stimulate mycotoxin production.

Using a liquid culture medium is an appropriate strategy for investigating the mechanisms of action of antifungal and antimycotoxin compounds. This approach promotes mycotoxin production and ensures close contact between fungal cells and bioactive molecules, making it suitable for evaluating their modes of action. Additionally, it enables comparisons of the effects of treatment on the exo- and endo-metabolomes of the pathogen grown under toxin-inducing and -repressing conditions [30,43]. However, when evaluating the effect of biomolecules/extracts on mycotoxin production, it is essential to consider the OTA levels quantified in the supernatants and in the mycelia. While *F. graminearum* cultivated in a liquid medium had mycotoxin levels in the supernatant that were over 4-fold higher than in the mycelium at the end of the culture period [43], *A. carbonarius* had high concentrations of OTA in the mycelium.

Our findings revealed that the efficacy of natural extracts, VIT, and fungicide in inhibiting fungal growth and OTA production varied depending on the concentration and the method used. Apart from tebuconazole, which inhibited fungal development, only VIT exhibited antifungal activity among the treatments tested. This effect was observed in liquid medium during the early stages of *A. carbonarius* development (2 and 3 dpi) but diminished at the later timepoints. The absence of fungal inhibition in solid medium and grape-berry assays, together with the progressive decline in inhibitory activity over time in liquid medium suggests that the concentrations tested were insufficient to achieve sustained suppression of fungal growth, particularly on solid substrates. This hypothesis is corroborated by the findings of Aznar et al. [29], who reported that higher concentrations of similar phenolic extracts (15–30 g/L) were required to achieve partial germination inhibition and strong growth inhibition in *A. flavus* growth. Consistent with their observations, our results with *A. carbonarius* growth on a solid medium show that doses insufficient to inhibit fungal development can nevertheless significantly modulate OTA production. All tested natural treatments significantly reduced OTA production in the solid medium and grape-berry assays. In liquid medium, Ta and VIT also significantly decreased total OTA accumulation, expressed as the sum of OTA concentrations measured in mycelia and culture supernatants at the end of the culture period (Figure S3). These results suggest that growth inhibition and mycotoxin suppression are uncoupled processes, likely governed by distinct mechanisms of action. In addition to the tested concentration, fungal adaptive responses, the chemical instability of the active compounds, and fungal detoxification mechanisms may also contribute to the reduced efficacy of the studied extracts/molecule, as suggested by Tran-Minh et al. [30]. Stilbenoids are known to be chemically unstable during storage and susceptible to isomerization and oxidation, particularly under aerobic conditions or when exposed to light [44]. Furthermore, *A. carbonarius* may activate detoxification pathways by expressing enzymes such as laccases, which are known to metabolize or inactivate phenolic compounds. For example, Taillis et al. [45] demonstrated that *Botrytis* laccase efficiently transformed or degraded certain stilbenoids, including resveratrol and the tetramer vitisin A. In addition, the tested products may induce the upregulation of fungal efflux pumps, such as ATP-binding cassette (ABC) or major facilitator superfamily (MFS) transporters, which are involved in the export of toxic compounds like stilbenoids, reducing their intracellular concentrations and mitigating their antifungal effects [46]. Taken together, these factors acting either individually or synergistically, may account for the moderate effect observed with the tested compound/extracts against *A. carbonarius* and could partially explain the gradual decrease in fungal growth inhibition over the course of the culture observed with VIT in liquid medium.

We observed that tebuconazole, a C_14_-demethylase inhibitor (DMI) that blocks ergosterol biosynthesis, led to growth inhibition and caused either a decrease (berry assay) or absence of inhibition, or even activation (solid medium) of OTA production. In fact, fungal and mycotoxin inhibition effects were not always positively correlated and varied depending on the method used and the product concentration. Several studies on *F. graminearum*, a trichothecene B producer and *F. proliferatum*, a fumonisin producer also reported increases in mycotoxin production or the induction of genes involved in mycotoxin biosynthesis following fungicide treatment [47,48,49] Regarding the use of fungicides against OTA producers, some studies on fungicide treatments showed no effect on OTA levels [50] while others reported stimulation of toxin production in *A. parasiticus*, *A. flavus*, and *A. carbonarius* at low doses [51,52,53]. The activation of OTA production in cultures treated with high levels of tebuconazole (0.16 g/L) could be explained by a direct stress effect on *A. carbonarius* increasing the activity of enzymes involved in toxin biosynthesis [47,54,55]. We observed a significant activation effect on intracellular OTA production in a liquid medium (OTA measured in biomass), particularly when Me extract was used. This extract was also found to upregulate the *otaA* and *otaC* OTA biosynthesis genes at 3 dpi (Figure 3B). Based on the results obtained in both liquid and solid media, Ta appears to be the most effective in inhibiting OTA production. This observation may be attributed to differences in stilbenoid composition. While both extracts contain comparable levels of VIT, the Ta extract is richer in stilbenoid monomers and oligomers (dimers and trimer). These compounds may contribute, either individually or through synergistic interactions, to the greater antimycotoxin activity observed for Ta.

Recent studies have proposed several hypotheses regarding the targets of stilbenoids which include the cell membrane, cell wall, mitochondria, DNA, cell division, the calmodulin calcineurin pathway, and the phosphoenolpyruvate-dependent phosphotransferase system [21,56]. However, regardless of the exact mechanisms by which mycotoxin production can be inhibited, it is not fully elucidated. Stilbenoids and OTA-producing fungi may interact through several indirect mechanisms. These include reducing OTA-induced toxicity, modulating OTA biosynthesis, and stimulating stilbenoid accumulation in plants [22,57]. In particular, Cai et al. [22] used molecular docking analyses to demonstrate that pterostilbene exhibits stronger binding affinities to several fungal target proteins than other tested stilbenes, which may explain its superior antifungal activity and ability to inhibit OTA production. The predicted targets included proteins involved in mitochondrial membrane function (P450), ergosterol biosynthesis (Erg24), and OTA biosynthesis (HAL and PKS), suggesting potential mechanisms through which stilbenes suppress fungal growth and mycotoxin production. Although the reduction in OTA production is generally attributed to the downregulation of genes involved in its biosynthetic pathway, our findings are consistent with those of Lappa et al. [58] who reported that certain *Lactobacillus plantarum* strains induced the upregulation of *AcOTAnrps* and *AcOTApks* despite significantly inhibiting mycotoxin production. The authors attribute these observations to the hypothesis that a different mode of action probably drives the anti-ochratoxigenic effect. Biological activity could induce a metabolic alteration, which is likely to affect expression of other genes leading to final protein expression. Post-transcriptional regulatory mechanisms may also be triggered by the significant upregulation of a biosynthetic pathway, which limits the translation of genes into proteins. Consequently, further research is required to understand the precise mechanism of action.

In a liquid medium, VIT and the stilbenoid-enriched extracts markedly reduced extracellular OTA accumulation despite only exerting a transient and limited effects on fungal growth. Surprisingly, this reduction was not systematically associated with decreased intracellular OTA levels and was accompanied by an early upregulation of OTA biosynthesis genes, suggesting that these compounds may trigger stress-response mechanisms rather than directly repress toxin biosynthesis. *A. carbonarius* initially perceives stilbenoids as a stress-inducing compounds and responds by transiently activating the OTA biosynthetic pathway as part of an adaptive response. One plausible explanation is that *Aspergillus* compensates for the inhibitory effect of VIT by transiently upregulating OTA biosynthetic genes. However, this response does not result in increased OTA accumulation, as toxin levels remained stable or declined from 3 to 4 dpi onward while gene overexpression progressively disappeared. This discrepancy suggests that VIT acts downstream of transcription, possibly through direct inhibition of enzymes involved in OTA biosynthesis. In particular, the antioxidant properties of VIT may impair the activity of *OtaC*, a cytochrome P450 enzyme essential for OTA production, either by altering cellular redox balance or through direct enzyme interaction. Alternatively, the reduction in extracellular OTA levels may result from impaired toxin export. As only the OTA concentration in the supernatants decreased following treatment, this observation may suggest an inhibition of either ATP-binding cassette (ABC)- or major facilitator superfamily (MFS)-type transporters, or that these transporters may also be involved in the efflux of compounds present in the extracts, thereby limiting OTA extrusion, possibly due to competitive interactions between molecules [46]. Polyphenols are known to modulate transporter activity through direct interactions with transport proteins, changes in ATPase activity, membrane fluidity, or transporter conformation. These effects appear to be highly dependent on the chemical structure of the polyphenol involved (e.g., resveratrol, catechins) [59].

These findings indicate that reduced OTA levels in culture supernatants may reflect altered toxin secretion, compartmentalization, or degradation rather than inhibition of biosynthesis pathways. However, it is possible that several post-transcriptional regulatory mechanisms exist that, in fine, limit OTA production. Further investigations, particularly focusing on key regulatory genes and OTA transport processes, are required to clarify the mode of action of stilbenoids and the mechanisms underlying OTA inhibition.

## 4. Conclusions

This study provides a wide evaluation of the effects of the stilbenoid-enriched vine root extracts, and the major stilbenoid, the tetramer VIT, on the growth and OTA production of *A. carbonarius*, using complementary liquid, solid, and ex vivo grape-berry assays. While each biological method has its own advantages and limitations, their combined use provided more robust and complementary information. In addition, our data showed that for accurate evaluation of biomolecules/extracts in liquid medium, OTA levels must be measured in both the supernatants and mycelia, since most of the toxin concentrates within the fungal biomass. Conversely, this issue does not arise in solid medium or grape-berries assay, where total mycotoxin content is quantified.

This study demonstrates the potential of vine by-products as a sustainable, value-added resources for managing OTA contamination in grapes. Furthermore, it highlights that the observed activities are driven by a complex mixture of compounds rather than individual molecules, suggesting that additive and/or synergistic effects occur between compounds of the same or different chemical groups. Further transcriptomics analyses are required to elucidate the molecular mechanism underlying the anti-OTA synergistic inhibition of these extracts.

To build upon these promising results, several avenues for future research should be explored. Evaluating these treatments against a panel of *A. carbonarius* strains is necessary to establish an objective, generalized assessment of their efficacy. In parallel, the stability, solubility, and bioavailability of the stilbenoid oligomers should be optimized through advanced and adapted formulation strategies, e.g., chitosan-based encapsulation. Furthermore, exploring combinations of these extracts with other bio-based matrices including lipids, alternative phenolic compounds, or essential oils that could uncover additive or synergistic effects, potentially enhancing their antifungal efficacy and avoiding the potential promycotoxigenic effects often observed at low doses. Despite the fact that resveratrol has GRAS (Generally Recognized As Safe) status and the safety of stilbenoids has been widely highlighted [21], further studies are needed to fully assess the safety of the stilbenoid-based product and its potential off-target effects on the phyllospheric microbiota.

## 5. Materials and Methods

### 5.1. Chemicals, Stilbenoid-Enriched Extracts, and Standards

For LC/FLD analysis, HPLC-grade acetonitrile from VWR (Fontenay-Sous-Bois, France) and acetic acid from Fisher Scientific (Loughborough, UK) were purchased while water was purified using an Elga water purification system (High Wycombe, UK). Reagents were purchased from Scharlau (Barcelona, Spain), Sigma Aldrich (St. Louis, MO, USA) and Fischer Scientific (Loughborough, UK).

Standard solution of OTA was purchased from Romer Labs (Tulln, Austria). The fungicide Corail^®^ containing the active molecule tebuconazole at a concentration of 250 g/L was purchased from Bayer (Lyon, France).

Vitisin B (VIT) (purity > 95% HPLC/DAD) was extracted and purified from grape-vine byproducts in the Molecules of Biological Interest Laboratory (ISVV, Villenave d’Ornon, France) following to the procedure described by Biais et al. [60]. Stilbenoid-enriched extracts were obtained from vine roots of Merlot and Tannat varieties in the Molecules of Biological Interest Laboratory, and their stilbenoid composition was characterized by Tardif et al. [27]. The concentrations of stilbenoids in the studied extracts are detailed in Table 1.

### 5.2. Plant Material and Preparation

Grape-berries of the Merlot variety were collected in September 2020 at the ripening stage from a plot of Grande Ferrade in Villenave d’Ornon (Latitude: 44.791742; Longitude: −0.580959), Nouvelle Aquitaine, France. Uninfected berries with intact cuticles were detached with cut pedicels, soaked in sterile water solution for 1 min, then in 70% ethanol (*v*:*v*) for 2 min, and finally rinsed with sterile water for 1 min. After disinfection, the berries were dried between sterile Whatman paper and placed on sand in sterile 12-well Nunc culture plates (Thermo Fisher Scientific, Loughborough, UK) with a diameter of 2 cm. A quantity of 1.5 g of Fontainebleau sand (size 180–500 µm, Carlo Erba reagents, Val-de-Reuil, France) was placed at the bottom of each well and moistened with 1 mL of sterile water. The berries were placed in the sand with the pedicel facing downwards.

### 5.3. Fungal Strain

*A. carbonarius* ATCC-4641 strain was used in this study, purchased from the American Type Culture Collection (ATCC) (reference: ATCC MYA4641—A1102 [ITEM 5010, IMI 388653] (Manassas, VA, USA), which was isolated from grape-berries in Brindis, Apulia, Italy.

The isolate was freeze-dried in a glass ampoule and its content was suspended in sterile water for 12 h. The spores were then deposited on two solid agar media: MEA (malt extract 20 g/L, Biokar, Allonne, France and agar 15 g/L, Sigma, Lezennes, France) and PDA (potato dextrose agar, Difco, Saint-Ferréol, France). After 10 days of incubation at 25 °C, the isolate was transferred by taking a 5 mm plug and deposited on a new solid medium. Dishes containing original strain was stored at 4 °C on MEA or PDA medium. When inoculum was required, the strain was grown on PDA slants at 25 °C in the dark for 5 days, after which spore suspensions were prepared by adding 6 mL of sterile distilled water to the PDA with gentle shaking. All transfers and experiments were carried out under a laminar flow hood.

The identity of the isolate was confirmed using PCR. After five days at 25 °C on MEA medium covered with a cellophane film (Hutchinsons, Chalette, Loing, France), the mycelium was scraped and DNA was extracted as described by Comont et al. [61]. Briefly, the freeze-dried mycelium was extracted with CTAB at 65 °C and one volume of chloroform/isoamyl alcohol (24:1, *v*:*v*). After centrifugation (10 min, 13,000 rpm, at 4 °C), the aqueous phase was re-extracted and the DNA precipitated overnight in 75% cold isopropanol at −20 °C. DNA was then dissolved in 50 µL of water and stored. Fungal identification was obtained by amplifying DNA with primers CARBO1 (AAG CGA ATC GAT AGT CCA CAA GAA TAC) and CARBO2 (TCT GGC AGA AGT TAA TAT CCG GTT), as described by Perrone at al. [62] using the following PCR parameters: denaturation at 95 °C for 2 min, followed by 35 cycles of 45 s at 90 °C, 50 s at 58 °C, and 1 min 30 s at 72 °C, followed by a final period of 10 min at 72 °C. The primers were specific to *A. carbonarius*.

### 5.4. Media and Culture Conditions

Liquid culture tests were performed in 24-well plates in CYA (Czapek Yeast Autolysate) medium: sucrose 30 g, yeast extract 5 g, NaNO_3_ 0.5 g, KCl 0.05 g, MgSO_4_·7H_2_O 0.5 g, FeSO_4_·7H_2_O 0.01 g, K_2_HPO_4_ 1.3 g, CuSO_4_·5H_2_O 0.005 g, ZnSO_4_·7H_2_O 0.01 g, distilled water 1000 mL. Each well contained 2 mL of CYA medium supplemented with either VIT (40 µM), Me (125 mg/L), Ta (125 mg/L), or 0.5% ethanol as a negative control. Each well was inoculated with 20 µL of an *A. carbonarius* ATCC-4641 conidial suspension at a concentration of 10^6^ conidia/mL, and the plates were incubated in darkness at 25 °C. Appropriate non-inoculated controls were performed with and without Me, Ta, and VIT. Fungal biomass and OTA production were quantified at 2, 3, 4, and 7 days post-inoculation (dpi). Following incubation, the cultures were centrifuged at 4816 *g* for 10 min. Supernatants were stored at −20 °C prior to OTA quantification. Fungal biomass was measured by weighing the mycelial pellet after lyophilization (48 h, Flexi-Dry, Oerlikon Leybold, Köln, Germany). Dry mycelia were stored at −20 °C until analysis. Biomass harvested at 2 and 3 dpi was kept at −80 °C for RNA extraction. Four replicates were performed for each condition.

For experiments in solid medium, the strain was grown on MEA at 4 °C for 5–7 days. The spores were collected in sterile water, counted using a hemocytometer and diluted to a concentration of 10^5^ spores/mL in sterile water containing 0.01% of Tween 80. Two main methods using spore suspensions were used on the MEA solid medium: in Method 1, one 300 µL drop was spread out (30 or 50 spores/mL) on the surface of the medium dishes (Figure S1). After drying and adsorption of spores on the surface of the medium, the product to be tested or ethanol 0.7% was sprayed in fine droplets onto the surface of the medium using a micro hand sprayer Ecospray (CXD, Fontenay-Sous-Bois, France). The Petri dishes were then sealed with cling film and placed in a growth chamber set to 25 °C with a 12/12 day/night cycle for six days. Method 2 involved mixing the spore suspension (30 or 50 spores/mL) directly with the product to be tested. The procedure was then the same as above, with drops being deposited and the dishes being incubated (Figure S1). Six days after inoculation, growth was assessed by measuring colony diameter (mm) when Method 2 was applied or by counting the number of colonies that had germinated on the Petri dishes when Method 1 was used.

For berries, the inoculation was carried out under sterile conditions. A sterile entomology needle (0.25 mm diameter) was used to create a wound at the top of each berry. Then, a 10 µL droplet of spore suspension (1200 or 2500 sp/mL) was applied (Figure S1). The spores were in either ethanol 0.7% or the product solution to be tested at the chosen concentration. The lids were placed on the boxes, which were then incubated in a growth chamber at 28 °C for 6 days. The negative control was treated with a drop of ethanol 0.7% and the positive control with a spore suspension without fungicide or stilbenoid extracts under the same conditions. To evaluate fungal development on the berries, a rating scale from 0 to 9, based on the extent of *A. carbonarius* development, was used (Figure S2). The results are the average of nine replicates concerning fungal development and three replicates concerning OTA quantification.

### 5.5. OTA Extraction and Quantification

For quantification of OTA in a sample of culture supernatant, 1 mL of the supernatant was filtered directly into vials for liquid chromatography coupled with a fluorescence detector (LC-FLD) analysis using a 0.2 µm syringe filter (Acrodisc 13 mm minispike PTFE, Thermo Fisher Scientific, Les Ulis, France).

OTA in mycelia was extracted by agitating the lyophilized ground mycelium with 10 mL of methanol for 60 min. After centrifugation (20 min at 3000 *g*), supernatants were evaporated to dryness under a nitrogen stream at 50 °C. The resulting residue was dissolved in 500 µL of acetonitrile-water (84:16, *v*/*v*) and filtered through a 0.2 µm filter prior to LC-FLD analysis.

For OTA quantification in solid media, 5 fungal plugs (0.5 cm of diameter) were suspended in 2 mL of methanol and sonicated for 15 min. The extracts were then centrifuged (15 min at 4816 *g*), after which 1.6 mL was evaporated to dryness under a nitrogen stream at 50 °C. The resulting residue was dissolved in 200 µL of acetonitrile-water (84:16, *v*/*v*) and filtered through a 0.2 µm filter prior to LC-FLD analysis.

For OTA extraction from berries, three frozen (−80 °C) berries were soaked in 21 mL of an acetonitrile/water MilliQ mix (84/16, *v*:*v*) and ground using an IKA Ultra Turrax^®^ T 25 digital (Staufen, Germany) at 16,000 r/min for 30 s. After centrifugation at 3000× *g* for 7 min, 7 mL were recovered and acidified with 70 µL of acetic acid and then purified on a MultiSep 229 Ochratoxin column (Romer Labs, Tulln, Austria). In total, 3 mL of the purified solution were evaporated to dryness under a nitrogen stream at 60 °C. The dry samples were dissolved in 300 µL of acetonitrile/water (84:16, *v*/*v*) filtered through 0.2 µm filters and transferred into vials for LC-FLD analysis.

Quantification of OTA was performed as described by Boonmee et al. [36] with slight modifications. A Shimadzu Prominence ultra-high-performance liquid chromatography coupled with fluorescence detector (UHPLC-FLD), equipped with two pumps LC-30 AD, a degasser DGU-20A5R, an auto sampler SIL-30 AC and a FLD detector RF-20A XS (Shimadzu Scientific Instruments, Noisiel, France) was used. Separation was achieved on a Kinetex 2,6U XB-C18—100Å column (150 × 4.6 mm; 2.6 µm) (Phenomenex, Le Pecq, France) maintained at 45 °C. The mobile phase consisted of 0.2% acetic acid in water (*v*/*v*) (solvent A) and acetonitrile (solvent B). The following gradient was used for elution: 45% B for 6 min, 45–95% B in 2 min, 95% B for 2 min, 95–45% B in 1 min, and 5 min post-run equilibration with initial conditions. The injection volume was 3 μL and the flow rate was kept at 1 mL/min. The fluorescence detector was set up at an excitation and emission wavelength of λex = 332 nm and λem = 466 nm. Quantification of OTA performed using an external calibration curve ranging from 0.005 to 5 µg/mL.

### 5.6. Extraction of Total RNA, Preparation of cDNA, and Real-Time RT-PCR Analysis

Mycelia collected at 2 and 3 days post-inoculation were homogenized in liquid nitrogen prior to RNA extraction using TRIzol reagent (Thermo Fisher, Courtaboeuf, France), following the manufacturer’s instructions. The RNA concentration was measured using a Quantus fluorometer (Promega, Leiden, The Netherlands). cDNA synthesis was performed using the iScript™ cDNA Synthesis Kit (Bio-Rad, Nazareth-De-Pinte, Belgium) and subsequently diluted fivefold with nuclease-free water.

RT-qPCR was performed on a QuantStudio™ 3 Real-Time PCR System (Applied Biosystems, Villebon-Sur-Yvette, France). Each reaction contained 6.25 µL of GoTaq^®^ qPCR Master Mix (Promega, Leiden, The Netherlands), 2 µL of cDNA, 0.625 µL of each primer (5 µM), and 2.492 µL of nuclease-free water. The thermal cycling conditions were as follows: initial denaturation at 95 °C for 3 min, followed by 39 cycles of 95 °C for 10 s and 60 °C for 30 s. A melting curve analysis was performed from 65 °C to 95 °C with an increment rate of 0.5 °C/s.

The primer sequences for all genes are listed in Table S1. Ubiquitin and Histone 3 were used as reference genes. Relative gene expression levels were calculated using the Ct method [63].

### 5.7. Expression of Results and Statistical Analyses

All growth measurements (fungal weight, colony diameter, and percentage of germinated spores) and OTA quantification data obtained from supernatants, mycelia, agar plugs or grape-berries were statistically analyzed using RStudio (version 4.3.3). Data normality was assessed using the Shapiro–Wilk test, and homogeneity of variances was evaluated using the Levene’s test, both at a significance level of 0.05. When assumptions of normality and homogeneity were met, parametric comparisons were performed using one-way ANOVA followed by Tukey’s HSD post hoc test. When these assumptions were violated, non-parametric analyses were carried out using the Kruskal–Wallis test followed by Dunn’s post hoc test. Level of significance was set at *p* = 0.05.

## Figures and Tables

**Table 1:** Stilbenoid content expressed in mg/g dry weight of stilbenoid-enriched extracts from Merlot and Tannat roots (adapted from Tardif et al. [27]).

Type of Stilbenoid Oligomer	Stilbenoid	Extract from Merlot Roots	Extract from Tannat Roots
Monomer	Resveratrol	8.3	21.2
	Piceatannol	ND	2.7
	**Total monomers**	**8.3**	**23.9**
Dimer	ε-Viniferin	7.1	24.6
	ω-Viniferin	ND	4.4
	Pallidol	ND	4.1
	Ampelopsin A	27.1	30.1
	**Total dimers**	**34.2**	**63.2**
Trimer	α-Viniferin	ND	ND
	Miyabenol C	ND	4.7
	**Total trimers**	**ND**	**4.7**
Tetramer	R-Viniferine (Vitisin B, VIT)	113.4	124.8
	**Total tetramers**	**113.4**	**124.8**
	**Total stilbenoids**	**155.9**	**216.6**

ND: not detected.

**Table 2:** Advantages and disadvantages of the testing methodologies used to evaluate the antifungal and antimycotoxin activity of vitisin B and stilbenoid-enriched extracts against *Aspergillus carbonarius*.

Methodologies	Advantages	Disadvantages
In vitro liquid medium	- Low cost - Easy to implement - Not time-consuming - Reproducibility - Accurate biomass and OTA quantification - Low consumption of the test product - Adapted for studying the mode of action of the test product on exo- and endo-metabolome	- To evaluate OTA production, it is necessary to quantify the toxin in both the culture supernatant and the mycelium. - Not representative of real conditions - Limited applicability to poorly water-soluble compounds
In vitro solid medium	- Low cost - Easy to implement - Not time-consuming - Reproducibility - More representative of real conditions than the method in a liquid medium - Administration of the tested product similar to real conditions	- High consumption of the tested product - Diffusion in the medium or adsorption - Difficult to quantify fungal biomass, necessitating the use of a cellophane film - Not adapted for studying the effect of the tested product on germination.
Ex vivo Grape-berry assay	- In vivo-like system, representative of real conditions - Administration of the tested product similar in real conditions	- Time-consuming - More complicated to implement - Seasonal dependance, need fresh untreated grape-berries - High consumption of the tested product - Difficult to quantify fungal biomass - Risk of contamination

## Data Availability

The original contributions presented in this study are included in the article and Supplementary Materials. Further inquiries can be directed to the corresponding authors.

## Supplementary Materials

The following supporting information can be downloaded at: https://www.mdpi.com/article/10.3390/toxins18090372/s1, Figure S1: Scheme illustrating the various inoculation and treatment techniques applied to *Aspergillus carbonarius* strain ATCC-4641 using (A) malt extract agar (MEA) medium and (B) Merlot berries as substrates. Figure S2: Rating scale used to evaluate the development of *Aspergillus carbonarius* on Merlot berries. Growth of *A. carbonarius* on detached grapes berries of Merlot at 28 °C, Isolate ATCC-4641, notation index of *A. carbonarius* development. Figure S3: Total ochratoxin A (OTA) accumulation by Aspergillus carbonarius ATCC 4641 at 7 dpi, supplemented with 40 µM (36.28 mg/L) vitisin B (VIT), 125 mg/L stilbenoid extract from Merlot roots (Me) or 125 mg/L stilbenoid extract from Tannat roots (Ta). Ethanol 0.5% was used as the control. Difference between control and treated conditions was determined using the Kruskal–Wallis test followed by Conever–Iman post hoc test. Different letters indicate a statistically significant difference between treatments (*p* < 0.05). Four biological replicates were performed. Total OTA represents the combined yield from mycelia and culture supernatant. dpi: days post inoculation. Table S1: Primers used for qPCR assays. Table S2: Effect of Merlot (Me) and Tannat (Ta) stilbenoid-enriched root extracts and of vitisin B (VIT) at 1 g/L on fungal development of ATCC-4641 strain compared to the corresponding control on the solid medium.

## Abbreviations

The following abbreviations are used in this manuscript:

C_14_-demethylase inhibitor	DMI
CTAB	Cetyltrimethylammonium bromide
CYA medium	Czapek Yeast Autolysate medium
FC	Fold change
GRAS	Generally Recognized As Safe
LC-FLD	Liquid chromatography coupled with a fluorescence detector
Me	Stilbenoid-enriched extract obtained from Merlot roots
MEA	malt extract agar
OTA	Ochratoxin A
Ta	Stilbenoid-enriched extract obtained from Tannat roots
UHPLC-FLD	Ultra-high-performance liquid chromatography coupled with fluorescence detector
VIT	Vitisin B
