# Supplementary material for: Efficacy of Stilbenoid-Enriched Vine Root Extracts in Controlling Aspergillus carbonarius Growth and Ochratoxin A Production in Grapes
Source: Toxins (Basel). 2026 Aug 29;18(9):372. doi: 10.3390/toxins18090372 (PMC13611447; doi:10.3390/toxins18090372)
Supplement: Supplementary file 1 [file toxins-18-00372-s001.zip › toxins-4460695-supplementary.pdf]

Supplementary Materials: Efficacy of Stilbenoid-Enriched Vine Root Extracts in Controlling *Aspergillus carbonarius* Growth and Ochratoxin A Production in Grapes

Trang Tran-Minh, Marie-France Corio-Costet, Marie Laurens, Nils Hocquemiller, Jessica Vallance, Marie-Noëlle Bonnin-Verdal, Pierre Waffo-Téguo, Florence Richard-Forget and Vessela Atanasova

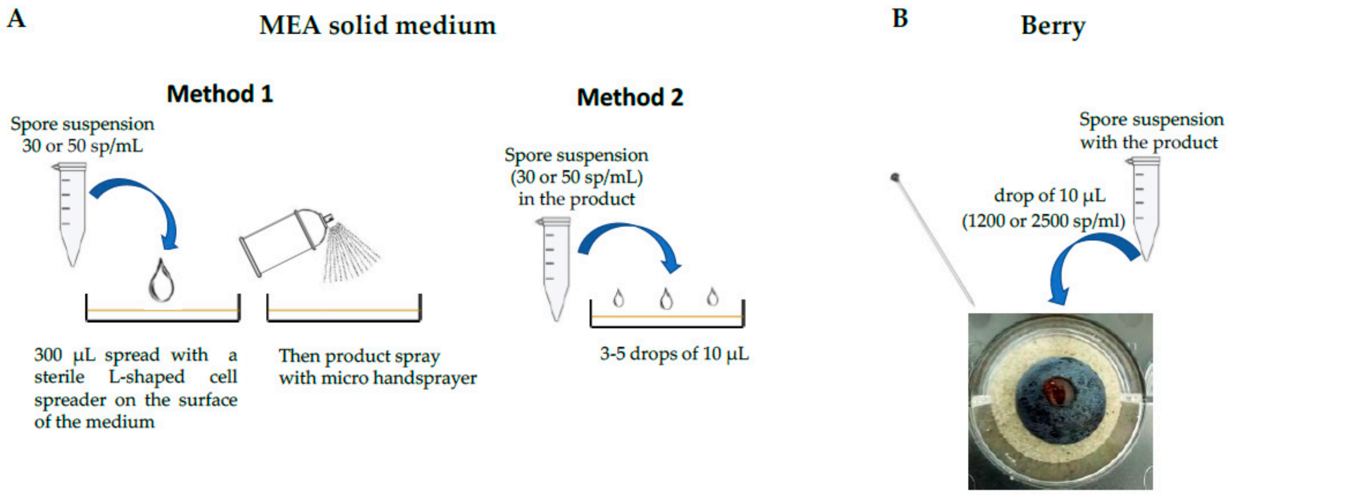

Figure S1. Scheme illustrating the various inoculation and treatment techniques applied to *Aspergillus carbonarius* strain ATCC-4641 using (A) malt extract agar (MEA) medium and (B) Merlot berries as substrates.

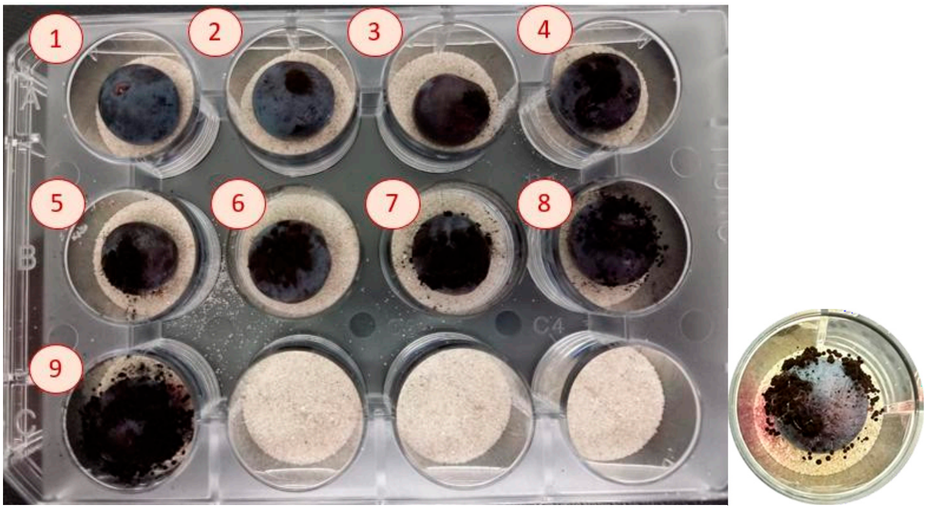

Figure S2. Rating scale used to evaluate the development of *Aspergillus carbonarius* on Merlot berries. Growth of *A. carbonarius* on detached grapes berries of Merlot at 28°C, Isolate ATCC-4641, notation index of *A. carbonarius* development.

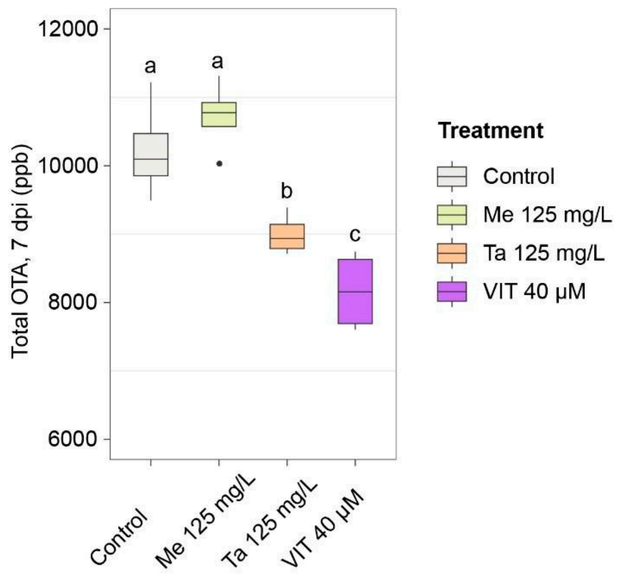

Figure S3. Total ochratoxin A (OTA) accumulation by *Aspergillus carbonarius* ATCC 4641 at 7 dpi, supplemented with 40 μM (36.28 mg/L) vitisin B (VIT), 125 mg/L stilbenoid extract from Merlot roots (Me) or 125 mg/L stilbenoid extract from Tannat roots (Ta). Ethanol 0.5% was used as the control. Difference between control and treated conditions was determined using the Kruskal–Wallis test followed by Conever–Iman post hoc test. Different letters indicate a statistically significant difference between treatments ($p < 0.05$). Four biological replicates were performed. Total OTA represents the combined yield from mycelia and culture supernatant. dpi: days post inoculation.

Table S1. Primers used for qPCR assays.

Gene ID	Gene annotation	Forward primer (5′-3′)	Reverse primer (5′-3′)	Efficiency (%)
ASPCADRAFT_399489	E2 ubiquitin-conjugating enzyme	GCTTCCAATCGAGCTA GACG	TAATGAC-CGCCTTCAT AGGG	84.2
ASPCADRAFT_177696	Histone H3	CCAGCGTTCTTACGAT CTCC	ATGGCGCAGA-GATTG GTATC	91.6
ASPCADRAFT_517149	Cytochrome P450 monooxy-genase (otaC)	CTTCTCCACGCTCGAC TACC	GCCGTGAAGCAG-TTA TAGGC	103.8
ASPCADRAFT_132610	Nonribosomal peptide syn-thetase (otaB)	ACAATTCCGTCCAGGT CAAG	ACTCAC-CACATCAACC AC-GA	103.3
ASPCADRAFT_173482	Highly reducing polyketide synthase (otaA)	ACAGTCTGCGCAATTC ACTG	ACTGCCGAG-TTCCTTC AAGA	110
ASPCADRAFT_209543	Halogenase (otaD)	TAATCCCATTGGCGAG TAGC	CCCAG-CAAGGATGTG AGAAT	85.6
ASPCADRAFT_209537	Probable cyclase (otaY)	TCCTCACCACCCTTGT CAAC	TATTCCCCTCGG-CAAT AACA	83.1

Table S2. Effect of Merlot (Me) and Tannat (Ta) stilbenoid-enriched root extracts and of vitisin B (VIT) at 1 g/L on fungal development[1] of ATCC-4641 strain compared to the corresponding control on the solid medium.

**Tested treatments**	**Control (number of germinated spores)**	**Treatment (number of germinated spores)**
Me	26.5 ± 7.1	26.7 ± 11.2
Ta	26.5 ± 7.1	26.7 ± 12.2
VIT	19.1 ± 5.3	17.1 ± 3.1

[1] Method 1 was used for the application of the spores and the treatments: the spores were spread on the solid medium and then sprayed with products or ethanol 0.7% for the control condition. For VIT, the data were based on three biological replicates, and for extracts, the data were based on four independent experiments, each performed in triplicate. Data are means ± standard deviation.
